# Real-world survival analysis by tumor mutational burden in non-small cell lung cancer: a multisite U.S. study

**DOI:** 10.18632/oncotarget.28178

**Published:** 2022-01-31

**Authors:** Connor Willis, Hillevi Bauer, Trang H. Au, Jyothi Menon, Sudhir Unni, Dao Tran, Zachary Rivers, Wallace Akerley, Matthew B. Schabath, Firas Badin, Ashley Sekhon, Malini Patel, Bing Xia, Beth Gustafson, John L. Villano, John-Michael Thomas, Solomon J. Lubinga, Michael A. Cantrell, Diana Brixner, David Stenehjem

**Affiliations:** ^1^Department of Pharmacotherapy, College of Pharmacy, University of Utah, Salt Lake City, UT, USA; ^2^Department of Pharmacy Practice and Pharmaceutical Sciences, College of Pharmacy, University of Minnesota, Duluth, MN, USA; ^3^Department of Internal Medicine, Huntsman Cancer Institute at the University of Utah, Salt Lake City, UT, USA; ^4^Department of Cancer Epidemiology, H. Lee Moffitt Cancer Center and Research Institute, Tampa, FL, USA; ^5^Department of Hematology and Oncology, Baptist Health Medical Group, Lexington, KY, USA; ^6^Department of Radiation Oncology, MetroHealth Medical Center, Cleveland, OH, USA; ^7^Division of Medical Oncology, Rutgers Cancer Institute of New Jersey, New Brunswick, NJ, USA; ^8^Department of Medicine, Kenneth Norris Jr. Comprehensive Cancer Center, University of Southern California, Los Angeles, CA, USA; ^9^Precision Oncology Program, Saint Luke’s Cancer Institute, Kansas City, MO, USA; ^10^Department of Internal Medicine, Markey Cancer Center, University of Kentucky, Lexington, KY, USA; ^11^US Medical Oncology, Bristol Myers Squibb, Princeton, NJ, USA; ^12^Health Economics and Outcomes Research, Bristol Myers Squibb, Princeton, NJ, USA; ^13^Global Medical Oncology, Bristol Myers Squibb, Princeton, NJ, USA

**Keywords:** lung neoplasma, tumor biomarkers, immunotherapy

## Abstract

Background: Tumor mutational burden (TMB) is a potential biomarker to predict tumor response to immuno-oncology agents in patients with metastatic non-small cell lung cancer (NSCLC).

Materials and Methods: A multi-site cohort study evaluated patients diagnosed with stage IV NSCLC between 2012 and 2019 who had received comprehensive genomic profiling (CGP) and any NSCLC-related treatment at 9 U.S. cancer centers. Baseline characteristics and clinical outcomes were compared between patients with TMB <10 and TMB ≥10.

Results: Among the 667 patients with CGP results, most patients received CGP from Foundation Medicine (64%) or Caris (20%). Patients with TMB ≥10 (vs. TMB <10) were associated with a positive smoking history. TMB was associated with ALK (*p* = 0.01), EGFR (*p* < 0.01), and TP53 (*p* < 0.05) alterations. TMB >10 showed a significant association towards longer overall survival (OS) (HR: 0.43, 95% CI: 0.21–0.88, *p* = 0.02) and progression-free survival (PFS) (HR: 0.43, 95% CI: 0.21–0.85, *p* = 0.02) in patients treated with first-line immunotherapy and tested by Foundation Medicine or Caris at treatment initiation.

Conclusions: TMB levels greater than or equal to 10 mut/Mb, when tested by Foundation Medicine or Caris at treatment initiation, were significantly associated with improved OS and PFS among patients treated with first-line immunotherapy-containing regimens. Additional prospective research is warranted to validate this biomarker along with PD-L1 expression.

## INTRODUCTION

Immune checkpoint inhibitors (ICIs) have substantially improved the clinical outcomes of some patients with metastatic non-small cell lung cancer (NSCLC) [1–4]. In late stage NSCLC, programmed death ligand 1 (PD-L1) expression by immunohistochemistry (IHC) is used in the clinical setting as a predictive biomarker as it may predict response to ICIs that target the programmed death receptor-1 (PD-1)/PD-L1 immune checkpoint pathway [4]. However, variations in measurement and interpretation of PD-L1 expression is a limitation to effectively compare PD-L1 expression across patients and tumor samples [5–6]. In addition, previous research has shown inconsistent results regarding the ability of PD-L1 expression to predict treatment response [2, 7–9]. These findings indicate a need to identify additional predictive biomarkers to select patients for immunotherapy. Improved patient selection would better identify patients who benefit from immunotherapy as well as spare patients predicted as non-responders from needless toxicity and cost. Tumor mutational burden (TMB) is a potential biomarker to predict a tumor’s sensitivity to immuno-oncology agents in a variety of advanced cancers [10]. In June 2020, The U.S. Food and Drug Administration (FDA) granted a tumor-agnostic indication for pembrolizumab as treatment for solid tumors with TMB >10 mut/Mb by FoundationOne CDx that have progressed following prior treatment and have no satisfactory alternative [11].

Tumors with higher TMB express more neoantigens – tumor-specific antigens that potentially allow for a more robust and durable immune response [12]. TMB can be measured by whole-exome sequencing (WES) and large targeted gene panels using next-generation sequencing (NGS) known as comprehensive genomic profiling (CGP). However, WES is currently associated with high cost and lengthy turnaround times, making it unsuitable for large scale and routine clinical applications [13, 14]. In contrast, CGP appears to be an effective and more efficient tool for TMB assessment in clinical practice. Several studies have demonstrated statistically significant correlation between TMB calculated from commercial targeted gene panels and from WES evaluations [13–15]. As a result, utilization of CGP in clinical practice is increasing, providing the opportunity for TMB evaluation to become a standard component of treatment decision making. In addition, label expansion for the use of immunotherapy in patients with high TMB levels in NSCLC and other tumor types is actively being explored [11]. The FDA has approved or authorized two targeted gene panels, FoundationOne CDx and MSK-IMPACT, for profiling solid tumors in clinical practice. CGP continues to evolve as technology improves and the number of companies offering TMB-enabled tests grows [16–18]. However, there is no standardized way to calculate a TMB score from a targeted gene panel. In addition, TMB levels from one testing panel are not interchangeable with those from another testing panel due to variations between tests. This includes the number of genes tested in the panel, the depth of sequencing, the types of mutations included, and the type of platform used; each company uses proprietary processes for testing and calculating TMB values [18–20]. Industry-wide efforts are underway to harmonize TMB results across platforms [21]. Therefore, questions remain regarding the clinical benefit of TMB testing when implemented across clinical practice.

In early reporting of TMB, a variety of different units and thresholds were used, including mutations per megabase (mut/Mb), percentiles, and total mutations [22]. Over time there has been a shift to the use of mut/Mb as the consensus unit for reporting TMB. The commonly used cut-off for TMB-high vs. TMB-low from tissue samples is >10 mut/Mb based on prospective testing of this threshold in NSCLC [11, 23–26]. Despite inconsistencies with TMB definition and reporting over time, high TMB has consistently been associated with improved clinical benefit among patients receiving immunotherapy for NSCLC [22]. We conducted a real-world multisite study to compare treatment response and survival outcomes among patients with metastatic NSCLC by TMB collected from tissue samples. The purpose of this study is to evaluate clinical outcomes by TMB among NSCLC patients treated with immunotherapy containing regimens in the first-line setting.

## RESULTS

**Figure 1:**
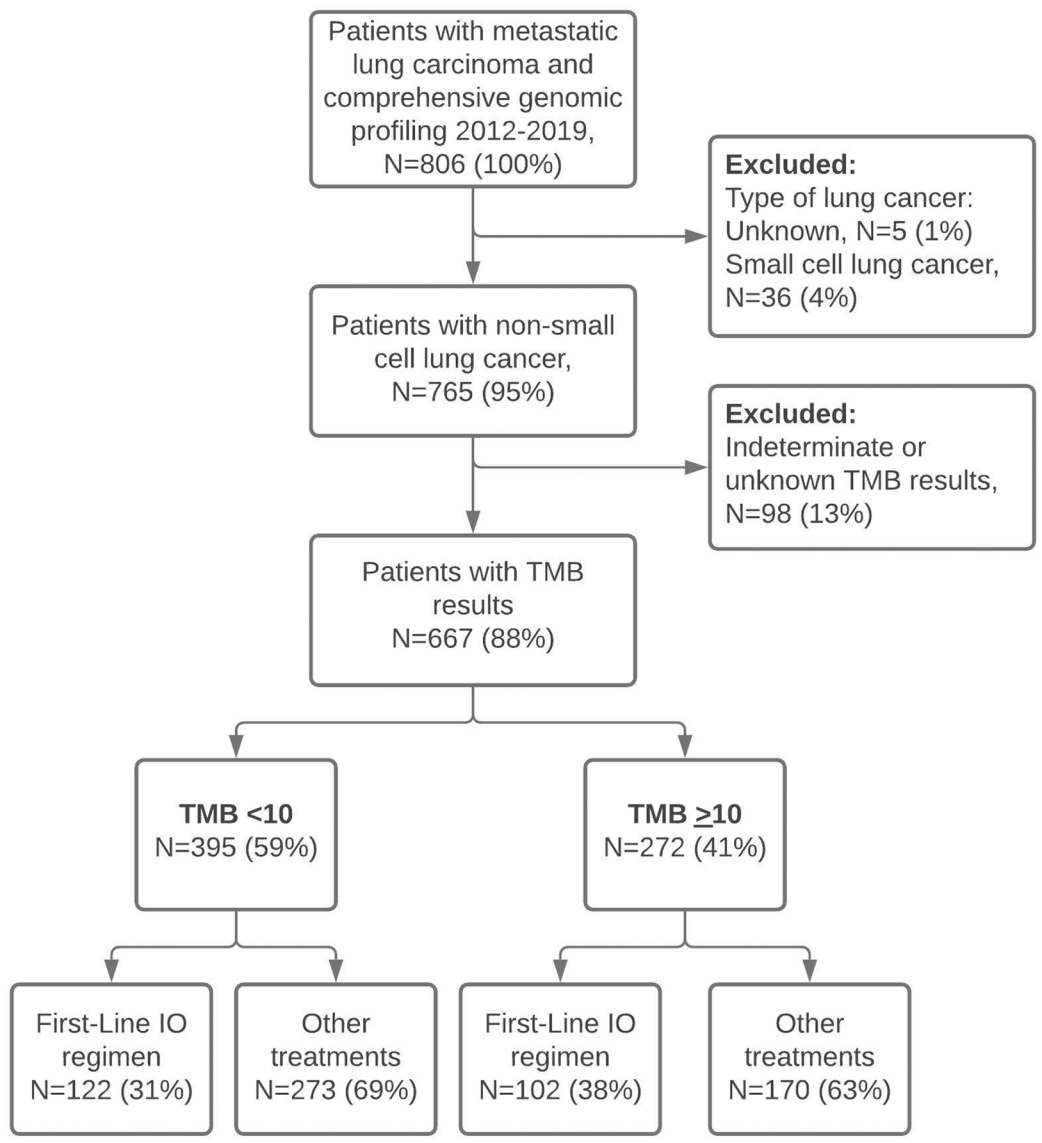
Study flow chart. Abbreviation: TMB: tumor mutational burden.

Nine U.S. cancer centers were selected to participate in the study. Five sites were members of the Oncology Research and Information Exchange Network (ORIEN) including H. Lee Moffitt Cancer Center & Research Institute (FL), Huntsman Cancer Institute at the University of Utah (UT), Rutgers Cancer Institute of New Jersey (NJ), Kenneth Norris Jr. Comprehensive Cancer Center at the University of Southern California (CA), and Markey Cancer Center at the University of Kentucky (KY). The four additional sites were MetroHealth Medical Center (OH), University of Washington (WA), Baptist Health System (KY), and Saint Luke’s Cancer Institute (MO). Sites were categorized by region, with the West region comprised of University of Washington, Huntsman Cancer Institute, and Kenneth Norris Jr. Comprehensive Cancer Center; the Central region included Markey Cancer Center, Baptist Health System, MetroHealth, and Saint Luke’s Cancer Institute; and the East region comprised of Rutgers Cancer Institute of New Jersey and H. Lee Moffitt Cancer Center & Research Institute. There were 765 patients who met study eligibility and 667 patients (88%) that had TMB results reported in mut/Mb (Figure 1). Of the 667 that met inclusion criteria, 204 patients (31%) were treated at community cancer centers compared to 463 (69%) patients treated at academic cancer centers.

**Figure 2:**
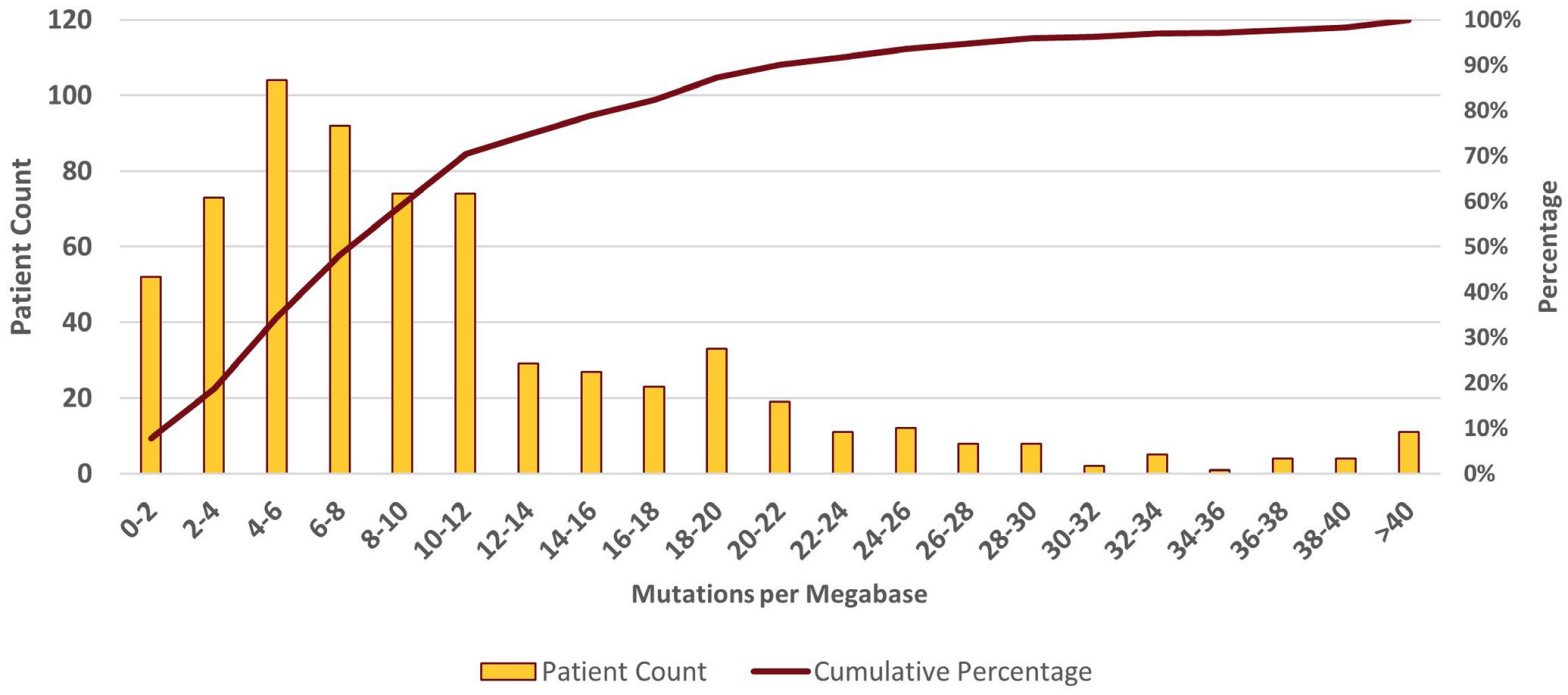
TMB distribution.

A total of 395 (59%) and 272 (41%) patients had low (<10) and high (>10) initial TMB measures, respectively (Figure 1). When using the Foundation Medicine classification thresholds, 34% were TMB-low (<6 mut/Mb), 53% were TMB-intermediate (TMB 6–19), and 13% were TMB-high (TMB >20). The median TMB value was 8 mut/Mb with an interquartile range (IQR) of 4–14 mut/Mb (Figure 2). The majority of patients received CGP from Foundation Medicine (64%), followed by Caris (20%), and Oncoplex (8%) (Supplementary Table 1). Foundation Medicine was widely utilized by six (67%) of the participating cancer centers, while Caris was utilized by two (22%) institutions, and all other testing platforms were utilized by a single institution.

### Demographic and clinical characteristics by TMB

Smoking status was significantly associated with TMB >10 with 91% of patients reported as current or former smokers compared to 61% in the TMB <10 cohort (*p* < 0.01, Table 1). Additionally, smoking status was associated with BMI as a higher proportion of smokers (includes secondhand smoke exposure) had BMI less than 18.5 kg/m^2^ or greater than 30 kg/m^2^.

**Table 1:** Demographic and clinical characteristics by tumor mutational burden

	TMB <10	TMB ≥10	Total	*P*-value
**Patient counts, *n* (%)**	395 (59)	272 (41)	667 (100)	
**Age, *n* (%)**				
<65 years	197 (50)	136 (50)	333 (50)	0.58^*^
65–74 years	129 (33)	96 (35)	225 (34)	
≥75 years	69 (17)	40 (15)	109 (16)	
Median age (IQR), years	65 (57–72)	65 (59–72)	65 (58–72)	0.37^†^
**Sex, *n* (%)**				
Female	228 (58)	141 (52)	369 (55)	0.13^*^
Male	167 (42)	131 (48)	298 (45)	
**Sex among ever smokers, *n* (%)**				
Female	139 (54)	125 (51)	264 (52)	0.49^*^
Male	119 (46)	121 (49)	240 (48)	
**Race, *n* (%)**				
White/Caucasian	299 (76)	205 (74)	504 (76)	0.32^§^
African American/Black	26 (7)	28 (10)	54 (8)	
Hispanic/Latino	16 (4)	12 (4)	28 (4)	
Asian	34 (9)	16 (6)	50 (8)	
Native American/American Indian	5 (1)	0 (0)	5 (1)	
Other	8 (2)	6 (2)	14 (2)	
Unknown	6 (2)	5 (2)	11 (2)	
**Body mass index (BMI)**				
Underweight (<18.5)	17 (5)	26 (10)	43 (7)	0.09^*^
Normal weight (18.5–24)	160 (45)	108 (42)	268 (43)	
Overweight (25–29)	105 (29)	70 (27)	175 (28)	
Obese (≥30)	77 (21)	56 (22)	133 (21)	
Median BMI (IQR)	25 (22–29)	25 (21–29)	25 (22–29)	0.13^†^
**PD-L1 expression, *n* (%)**				
<1%	113 (41)	75 (36)	188 (39)	0.11^*^
1–49%	87 (32)	57 (28)	144 (30)	
≥50%	75 (27)	75 (36)	150 (31)	
**Smoking history, *n* (%)**				
Current smoker	41 (10)	83 (31)	124 (19)	**<0.01**^§^
Former smoker	201 (51)	162 (60)	363 (54)	
Never smoker	135 (34)	21 (8)	156 (23)	
Passive (second-hand) smoke	16 (4)	1 (0)	17 (3)	
Unknown	2 (1)	5 (2)	7 (1)	
Median pack-years smoke (IQR)	30 (15–45)	40 (25–50)	35 (20–49)	**<0.01**^†^
**Comorbidities, *n* (%)**^‡^				
Asthma	31 (8)	14 (5)	45 (7)	0.17^*^
Cerebrovascular diseases	15 (4)	23 (8)	38 (6)	0.01^*^
Diabetes	62 (16)	50 (18)	112 (17)	0.36^*^
Hepatitis C	6 (2)	8 (3)	14 (2)	0.21^*^
Myocardial infarction	10 (3)	13 (5)	23 (3)	0.12^*^
Peripheral vascular disease	12 (3)	16 (6)	28 (4)	0.07^*^
Pneumonia	47 (12)	32 (12)	79 (12)	0.96^*^
Pulmonary disease	74 (19)	81 (30)	155 (23)	<0.01^*^
**ECOG PS at diagnosis, *n* (%)**				
0	80 (20)	32 (12)	112 (17)	0.02^*^
1	153 (39)	112 (41)	265 (40)	
2+	54 (14)	49 (18)	103 (15)	
Unknown	108 (27)	79 (29)	187 (28)	
**Histology subtypes, *n* (%)**				
Adenocarcinoma	327 (84)	214 (79)	541 (82)	0.35^*^
Squamous cell carcinoma	38 (10)	36 (13)	74 (11)	
Large cell carcinoma	7 (2)	8 (3)	15 (2)	
Not otherwise specified	19 (5)	12 (4)	31 (5)	
**Stage at metastatic diagnosis, *n* (%)**				
IV	170 (43)	130 (48)	300 (45)	0.25^*^
IVA	143 (37)	99 (37)	242 (37)	
IVB	78 (20)	41 (15)	119 (18)	
**Sites of metastases, *n* (%)**^‡^				
Brain	153 (39)	112 (41)	265 (40)	0.53^*^
Bone	196 (50)	100 (37)	296 (44)	<0.01^*^
Liver	79 (20)	43 (16)	122 (18)	0.17^*^
Lymph	63 (16)	57 (21)	120 (18)	0.10^*^
Other	174 (44)	91 (33)	265 (40)	0.01^*^

^*^Chi square test, ^§^Fisher’s Exact, ^†^Wilcoxon rank-sum. Abbreviations: TMB: Tumor mutational burden; *p*-value: probability value; IQR: interquartile range; BMI: body mass index; ECOG PS: Eastern Cooperative Oncology Group Performance Status; Ever smokers include exposure to second-hand smoke. Biopsy for TMB testing taken from primary tumor. Metastatic data gathered from initial diagnosis. ^‡^Comorbidities and sites of metastases are not mutually exclusive.

The distribution of Eastern Cooperative Oncology Group performance status (ECOG PS) was significantly associated with TMB – an ECOG PS of >2 was seen in 18% of patients with TMB >10 vs. 14% of TMB <10 (*p* = 0.02, Table 1). However, the association was not statistically significant when controlling for smoking history and BMI (*p* = 0.10). Another significant association was observed between TMB and bone metastases. Patients with TMB <10 showed a higher prevalence of bone metastases compared to patients with TMB >10 (*p* < 0.01, Table 1).

No association was seen between TMB and age, tumor histology, or cancer stage at diagnosis (Table 1). Additionally, there was no statistically significant difference in distribution of sex between cohorts (*p* = 0.13, Table 1). Sex was not associated with TMB even when controlling for smoking history (*p* = 0.49, Table 1).

The median (IQR) TMB among patients with PD-L1 <1% was 8 mut/Mb (5–14.5), 7.5 (4–13) for patients with PD-L1 1–49%, and 9.5 mut/Mb (5–15) for patients with PD-L1 >50%. No association was found between TMB distribution and the PD-L1 categories (*p* = 0.32). In addition, evaluation of PD-L1 expression among patients with TMB <10 and TMB >10 showed no association (*p* = 0.11, Table 1).

### TMB testing patterns

The median (IQR) duration from diagnosis of metastatic NSCLC to CGP testing was 38 days (16–162). The median duration from diagnosis to CGP testing was significantly shorter among community vs. academic cancer centers (20 days vs. 52 days, *p* < 0.01). The biopsy taken on the day of diagnosis was used for CGP testing in 41% of patients. The median duration from CGP testing to initiation of systemic therapy was seven days (IQR: -147-27); a negative value represents a patient that initiated systemic therapy for metastatic disease prior to CGP testing. The median duration from ordering CGP to the reception of test results was 14 days (IQR: 11–19) and the majority of patients initiated systemic therapy prior to the receipt of CGP results. The majority of patients (63%) received CGP testing within 60 days of treatment initiation (Supplementary Figure 1).

### Association of TMB with specific genetic alterations

Lower TMB was associated with *ALK* (median TMB = 5 for *ALK* alterations vs. TMB = 8 for *ALK* wild-type, *p* = 0.01) and *EGFR* (median TMB = 5 for *EGFR* alterations vs. TMB = 9 for *EGFR* wild-type, *p* < 0.01) alterations (Table 2). Higher TMB was associated with *TP53* alterations (median TMB = 10 for *TP53* alterations vs. TMB = 6 for *TP53* wild-type, *p* < 0.01) (Table 2). Actionable genomic mutations that were not significantly associated with TMB included *BRAF* (*p* = 0.18), *ROS1* (*p* = 0.24), and *RET* (*p* = 0.43).

**Table 2:** Significant associations between tumor mutational burden and genomic alterations

Genomic Alteration	Number with alterations, (%)		Number tested for each gene alteration, (%)	*p*-value^*^	Median (IQR) TMB (mut/Mb)		*p*-value^†^	Adj *p*-value^§^
	TMB <10	TMB ≥10			Gene alteration	Wild-type		
**ALK**	41 (10)	18 (7)	662 (87)	0.09	5 (3–11)	8 (4–14)	**<0.01**	**0.01**
**AR**	69 (21)	73 (35)	539 (70)	**<0.01**	10 (5–18)	6.5 (3.5–11)	**<0.01**	**<0.01**
**ARID1A**	16 (5)	24 (11)	540 (71)	**<0.01**	10.5 (7–14)	7 (4–13)	**0.01**	**0.03**
**EGFR**	128 (33)	38 (14)	663 (87)	**<0.01**	5 (3–9)	9 (5–16)	**<0.01**	**<0.01**
**KEAP1**	14 (4)	22 (9)	589 (77)	**0.03**	12 (8–20)	8 (4–14)	**<0.01**	**0.01**
**LRP1B**	20 (7)	38 (15)	552 (72)	**<0.01**	15 (8–22)	8 (4–14)	**<0.01**	**<0.01**
**MLH1**	5 (1)	14 (5)	652 (85)	**<0.01**	16 (7–21)	8 (4–13)	**<0.01**	**0.01**
**MPL**	38 (12)	55 (26)	538 (70)	**<0.01**	12 (7–20)	6 (4–11)	**<0.01**	**<0.01**
**MSH2**	5 (1)	14 (5)	661 (86)	**<0.01**	14 (7–19)	8 (4–13)	**<0.01**	**0.01**
**MSH6**	7 (2)	15 (6)	652 (85)	**0.01**	11.5 (7–19)	8 (4–14)	**0.01**	**0.03**
**NF1**	19 (5)	25 (9)	653 (85)	**0.03**	11.5 (7–18)	8 (4–13)	**<0.01**	**0.01**
**NTRK3**	3 (1)	9 (3)	661 (86)	**0.02**	19 (10.5–19.5)	8 (4–13)	**<0.01**	**0.01**
**PTEN**	40 (10)	44 (16)	653 (85)	**0.03**	10.5 (6–16)	8 (4–13)	**<0.01**	**0.01**
**SMARCA4**	14 (4)	20 (10)	540 (71)	**0.01**	10 (6–18)	7 (4–12.5)	**<0.01**	**0.01**
**SPTA1**	16 (6)	23 (12)	465 (61)	**0.02**	10 (8–18)	7 (4–14)	**0.01**	**0.02**
**STK11**	36 (9)	61 (23)	653 (85)	**<0.01**	11 (6–18)	7 (4–13)	**<0.01**	**<0.01**
**TP53**	187 (49)	206 (77)	654 (85)	**<0.01**	10 (6–18)	6 (3–9)	**<0.01**	**<0.01**

^*^Chi square test, ^†^Wilcoxon Rank Sum Test, ^§^Benjamini-Hochberg procedure. Abbreviations: TMB: Tumor mutational burden; *p*-value: probability value; IQR: interquartile range. Genes that were not associated with TMB but had a frequency of ≥5% in this study population: *AKT1, ATM, BRAF, CDKN2A, CDKN2B, ERBB2, FOXP1, KDM5C, KRAS, MET, MYC, NF2, NRAS, NTRK1, NTRK2, PIK3CA, PMS2, POLD1, POLE, RB1, RMB10, RET, RICTOR, ROS1.*

### Treatment patterns and treatment response for first-line immunotherapy containing regimens

In the first-line setting, an immunotherapy-containing regimen was received by 38% (*n* = 102) of patients with TMB >10 and 31% (*n* = 122) of patients with TMB <10. An immunotherapy-containing regimen included both immunotherapy monotherapy and combination chemoimmunotherapy. The proportion of patients who received first-line immunotherapy grew consistently from 18% in 2014 to 76% in 2019, while the proportion of patients who received only chemotherapy first-line consistently decreased from 50% in 2014 to 10% in 2019 (Supplementary Figure 2). Of patients with PD-L1 <1%, 45% received an immunotherapy-containing regimen first-line compared to 56% and 54% for patients with PD-L1 1–49% and PD-L1 >50%, respectively (Supplementary Figure 3).

In patients who received an immunotherapy-containing regimen first line and CGP testing by Foundation Medicine or Caris, overall response rate was observed in 38% of patients with TMB <10 and 35% of patients with TMB >10 (*p* = 0.87).

### Overall survival for first-line immunotherapy containing regimens

A multivariable model was used to analyze OS by TMB. The median follow-up time for patients included in the model was 9.0 months. The multivariable model controlled for age, cancer stage, ECOG PS, histology, smoking status, first-line treatment regimen, testing platform, region of cancer center, and PD-L1 expression. Only patients who received a first-line immunotherapy-containing regimen and testing by Foundation Medicine or Caris were included in the model (*n* = 206). Compared to patients with TMB <10, OS was longer for patients with TMB >10, but was not statistically significant (HR: 0.55, 95% CI: 0.28–1.05, *p* = 0.07) (Table 3). At end of follow-up, 67% and 68% of patients with TMB <10 and TMB >10 were alive, respectively. The subgroup analysis of patients who received TMB testing within 60 days of treatment initiation (*n* = 141) demonstrated significantly longer OS for patients with TMB >10 (HR: 0.43, 95% CI: 0.21–0.88, *p* = 0.02), compared to their TMB <10 counterparts. Of the 141 patients who received TMB testing within 60 days of treatment initiation, the biopsy used for TMB testing was taken prior to treatment initiation in 97%.

**Table 3:** Overall survival by tumor mutational burden and other relevant variables: univariable and multivariable model

Parameter	*n*	# of deaths	Univariable			Multivariable		
			HR	95% CI	*p*-value	HR	95% CI	*p*-value
TMB (≥10 vs. <10 mut/Mb)	184	58	1.03	0.62–1.74	0.89	0.55	0.29–1.05	0.07
PD-L1 (1–49% vs. <1%)	95	29	0.84	0.41–1.76	0.66	0.65	0.28–1.50	0.31
PD-L1 (≥50% vs. <1%)	98	37	1.19	0.61–2.35	0.61	0.72	0.30–1.70	0.45
PD-L1 (1–49% vs. ≥50%)	115	40	0.71	0.38–1.34	0.29	0.91	0.40–2.08	0.82
Stage (IVa vs. IVb)	142	37	0.78	0.38–1.59	0.49	0.48	0.22–1.08	0.08
ECOG PS (0 vs. 1)	110	35	0.71	0.34–1.49	0.36	0.55	0.24–1.25	0.15
ECOG PS (0 vs. ≥2)	62	25	0.23	0.10–0.53	<0.01	0.13	0.05–0.36	<0.01
Age (<75 vs. ≥75)	206	67	0.44	0.26–0.78	<0.01	0.39	0.19–0.80	0.01
Never Smoked vs. Smoked	203	66	1.32	0.74–2.37	0.34	0.71	0.34–1.48	0.36
Squamous vs. Non-squamous	201	66	0.78	0.33–1.80	0.56	0.50	0.14–1.79	0.29
IO + Chemo vs. IO monotherapy	206	67	0.71	0.44–1.17	0.18	0.64	0.31–1.30	0.21

Includes patients treated with first-line immunotherapy and TMB testing by Foundation Medicine or Caris; Multivariable model accounts for TMB test and the region of cancer institute. Abbreviations: TMB: Tumor mutational burden; OS: overall survival; ECOG PS: Eastern Cooperative Oncology Group Performance Status; Smoked includes exposure to second hand smoke.

Additionally, an exploratory analysis demonstrated that when TMB was included in the multivariable model as a continuous variable (per unit change) (*n* = 191), higher TMB was associated with improved survival (HR: 0.94, 95% CI: 0.89–0.98, *p* = 0.01).

### Progression-free survival for first-line immunotherapy containing regimens

A multivariable model was used to analyze PFS by TMB. The median number of days in PFS (measured from initiation of first-line immunotherapy to physician-documented progression, or death) was longer for patients with TMB >10 (388 days) than TMB <10 (203 days). The multivariable model controlled for age, cancer stage, ECOG PS, testing platform, histology, smoking history, treatment regimen, region of cancer center and PD-L1 expression. Only patients who received a first-line immunotherapy-containing regimen, were tested by Foundation Medicine or Caris, and had available date of disease of progression data were included in the model (*n* = 200). TMB >10 was associated with increased PFS (HR: 0.36, 95% CI: 0.21–0.65, *p* < 0.01) (Table 4). The subgroup analysis of patients who received TMB testing within 60 days of treatment initiation (*n* = 141) also demonstrated longer PFS for patients with TMB >10 (HR: 0.43, 95% CI: 0.21–0.85, *p* = 0.02), compared to their TMB <10 counterparts.

**Table 4:** Progression-free survival univariable and multivariable model

Parameter	*n*	# of events	Univariable			Multivariable		
			HR	95% CI	*p*-value	HR	95% CI	*p*-value
TMB (≥10 vs. <10 mut/Mb)	179	75	0.76	0.48–1.21	0.25	0.36	0.21–0.65	<0.01
PD-L1 (1–49% vs. <1%)	94	38	0.53	0.28–1.01	0.06	0.62	0.30–1.29	0.20
PD-L1 (≥50% vs. <1%)	96	50	0.53	0.29–0.97	0.04	0.63	0.29–1.36	0.24
PD-L1 (1–49% vs. ≥50%)	112	50	1.00	0.56–1.79	0.99	0.98	0.47–2.05	0.96
Stage (IVa vs. IVb)	137	56	0.51	0.29–0.89	0.02	0.35	0.19–0.67	<0.01
ECOG PS (0 vs. 1)	112	47	0.90	0.49–1.65	0.73	0.67	0.33–1.37	0.27
ECOG PS (0 vs. ≥2)	60	32	0.43	0.21–0.88	0.02	0.32	0.13–0.78	0.01
Age (<75 vs. ≥75)	200	88	0.76	0.42–1.38	0.36	0.47	0.23–0.96	0.04
Never Smoked vs. Smoked	197	86	1.18	0.73–1.91	0.50	0.47	0.26–0.86	0.01
Squamous vs. Non-squamous	200	88	1.12	0.54–2.33	0.76	0.45	0.17–1.23	0.12
IO + Chemo vs. IO monotherapy	200	88	0.89	0.56–1.40	0.60	0.76	0.39–1.45	0.40

Includes patients treated with first-line immunotherapy and TMB testing by Foundation Medicine or Caris; Multivariable model accounts for TMB test and the region of cancer institute. Abbreviations: TMB: Tumor mutational burden; OS: overall survival; ECOG PS: Eastern Cooperative Oncology Group Performance Status; Smoked includes exposure to secondhand smoke.

Additionally, an exploratory analysis demonstrated that longer PFS was also associated with higher TMB, when TMB was assessed as a continuous variable (per unit change) (HR: 0.96, 95% CI: 0.92–0.99, *p* = 0.01) (*n* = 189).

PFS was significantly longer when patients with TMB >10 were treated with an immunotherapy-containing regimen first-line compared to first-line therapy of chemotherapy (HR: 0.55, 95% CI: 0.34–0.88, *p* = 0.01) (Supplementary Figure 4).

## DISCUSSION

This study evaluated two broad questions: (1) The distribution of TMB in the real world and its association with baseline clinical and demographic features (*n* = 677) and (2) the association between TMB and clinical outcomes among NSCLC patients who received first-line immunotherapy (*n* = 224). The distribution of TMB in this cohort was right skewed with 41% of patients over 10 mut/Mb, 13% over 20 mut/Mb, and a median value of 8 mut/Mb. The distribution of TMB in this study matches closely with published data from the FoundationCORE database [27]. In contrast with previous research, this study did not show an association between sex and TMB even when accounting for smoking history. However, previous research showing associations between males and higher TMB levels were conducted in other populations with different methodologies and smaller sample sizes [22, 28–30]. Previous research is inconclusive regarding an association between higher TMB and older patients [22, 29–31]. This study did not show an association between age and TMB. A positive smoking history has consistently been shown to correlate with higher TMB levels [13, 22, 28, 30–32]. This study confirmed the association between a positive smoking history and TMB >10. The association between higher TMB and tumor histology has been explored previously with mixed results [22, 28, 31]. No association between histology and TMB was observed in this cohort. The relationship between histology and TMB requires additional testing.

This study, conducted across 8 academic cancer centers, found no association between TMB and PD-L1 expression in concordance with a majority of previous studies [2, 22, 32–35]. This study also confirmed results from previous research that has consistently shown an association between *EGFR* alterations and low TMB [22, 25]. Tumors with driver mutations may have lower overall levels of genomic instability leading to the lower levels of TMB seen in patients with *EGFR* and *ALK* alterations. The association between *TP53* alterations and higher TMB also confirms previous research results [22, 25].

Multivariable models, controlling for confounding variables, were utilized to assess the impact of TMB on clinical outcomes. One source of confounding is the variation in measured TMB between testing platforms. Therefore, only patients tested by Foundation Medicine and Caris were included in the model. Foundation Medicine and Caris were selected because the median (IQR) TMB levels of Foundation Medicine: 8 (4–15), and Caris: 10 (7–15) were similar, and the two platforms account for 84% of the patients in this study cohort.

Previous evaluations in lung carcinomas have shown a general trend towards increased OS in patients with higher TMB [2, 32, 36–43]; however, few studies have reported a statistically significant association between TMB and OS [37, 42]. The primary OS analysis demonstrated a statistically non-significant trend towards improved OS and higher levels of TMB in patients treated with ICIs. However, when assessing OS by TMB in patients with TMB testing within 60 days of treatment initiation the association became statistically significant. These results suggest an association between higher TMB and increased OS, with the greatest predictive value when TMB is assessed at treatment initiation, possibly due to TMB changes with time and treatment. Interestingly this model did not show increased OS for ICI-treated smokers vs. non-smokers while controlling for TMB levels. This suggests that previously reported associations between positive smoking status and increased survival when treated with ICI may be explained by increased TMB in these patients [44].

Published literature shows a consistent association between higher TMB and increased PFS in lung carcinomas, with several studies reporting a significant association [13, 32, 36, 39, 45, 46]. An increase in PFS for patients with TMB >10 treated with first-line immunotherapy was observed in this study. Additionally, immunotherapy-containing regimens resulted in superior PFS when compared to chemotherapy providing evidence for TMB as a predictive biomarker for first-line treatment.

In contrast with previous work, this study did not show an association between high TMB and improved objective response rate [11, 13, 22, 43]. However, due to the retrospective nature of this study design, there were significant limitations associated with accurately assessing treatment response based on physician documentation across institutions. Higher weight should be given to results from clinical trials with access to pathologic images that allow for consistent evaluations of objective response rate according to RECIST criteria.

In response to the published data suggesting an association between TMB and clinical outcomes, several manufacturers of PD-(L)1 inhibitors have submitted supplemental biologic license applications to the FDA. The FDA granted a tumor-agnostic indication for pembrolizumab in June 2020, as treatment for patients with TMB >10 mut/Mb by FoundationOne CDx whose disease have progressed following prior treatment across solid tumors and who have no satisfactory alternative [11]. The approval was based on results from the KEYNOTE-158 trial which assessed patients with a variety of tumor types, including anal, biliary, cervical, endometrial, mesothelioma, neuroendocrine, salivary, small cell lung, thyroid, and vulvar cancers [11]. This trial reported an objective response rate of 29% in patients with TMB-high vs. 6% in patients with TMB-low [11]. Multiple retrospective analyses of pembrolizumab by TMB levels have sought to verify results from the KEYNOTE-158 in the NSCLC population with varying results. A combination analysis of the KEYNOTE-010 and KEYNOTE-042 cohorts demonstrated increased clinical efficacy in patients treated with pembrolizumab vs. chemotherapy in patients with TMB >175 mutations per exome; whereas, the exploratory analyses in KEYNOTE-021G, KEYNOTE-189, and KEYNOTE-407 cohorts of combination pembrolizumab plus chemotherapy showed no association between TMB and clinical outcomes [47].

These results show that there is a significant proportion of patients with PD-L1 levels <1% that are receiving first-line immunotherapy (23%) (Supplementary Figure 3). As shown in Table 4, immunotherapy containing regimens have improved PFS independent of PD-L1 levels when given to patients with TMB levels > 10 mut/Mb. Therefore, patients with PD-L1 <1% that have high TMB may achieve improved clinical outcomes and be spared of harmful side effects of cytotoxic chemotherapy agents.

The evaluation of additional PD-(L)1 inhibitors in patients with TMB-high NSCLC is actively being investigated. In the NEPTUNE study, a randomized, phase III study of durvalumab plus tremelimumab compared to chemotherapy in patients with stage IV NSCLC, no difference in OS was observed in patients with blood based TMB (bTMB) >20 mut/Mb [48]. In the MYSTIC study, another phase III study, an exploratory analysis of durvalumab plus tremelimumab showed improvements in OS, PFS, and objective response rate compared to chemotherapy in patients with NSCLC with bTMB >20 with a trend for improved survival with TMB >10 from tissue samples [49]. The B-F1RST study, a phase II study of atezolizumab in first-line NSCLC demonstrated improved PFS in the bTMB >16 cohort vs. the bTMB <16 cohort (5.0 vs. 3.5 months) [50].

This study focused specifically on TMB determined from tissue samples. The decision to exclude liquid (blood) TMB biopsies was based on the lack of existing evidence to support bTMB biopsies at the time the study was initiated. As more bTMB tests enter the market, future studies would benefit from collecting all TMB test results and stratifying them by type of biopsy.

The results of this study provide a comprehensive real-world assessment of clinical outcomes by TMB across cancer centers in the U.S. By including both academic and community cancer centers from various geographic regions in the U.S., we were able to provide a representative dataset with all cancer centers contributing 6–20% of the total sample size.

### Limitations

TMB testing is not standardized between testing vendors; therefore, inclusion of multiple testing platforms may introduce bias in the results. In addition, the threshold of 10 mut/Mb may not be the optimal cut-off for each test or the best cut-off for NSCLC. Therefore, clinical outcomes were assessed in a subgroup of patients that received TMB testing from either of the two most commonly used vendors, Foundation Medicine (*n* = 491) or Caris (*n* = 152). Differences in TMB levels across these two testing vendors were not seen (*p* = 0.73) and by limiting the testing platforms included in the clinical outcome analyses we were able to limit the potential bias from differences in TMB levels by testing platform.

Because of the retrospective nature of this study design, disease progression and response data were collected by chart review and did not follow RECIST criteria. Additionally, medical charts and tumor registries may be subject to missing data and coding errors. The specific type and location of genomic alterations was not captured, and patients were not prospectively treated according to TMB. Although TMB was predominantly ascertained around treatment initiation, 37% of TMB measurements were obtained outside of 60 days from treatment initiation and where therefore not included in the OS/PFS subgroup analyses. Smoking status was used as a crucial predictor of both TMB and clinical outcomes, but was not able to quantify the degree of smoking exposure. Pack-years was not used due to the number of missing results across the cohort.

This study includes only patients who had their tumor samples submitted for CGP, and these results may not be generalizable to patients whose tumors are never tested by CGP. Additionally, because not all patients were tested and in the earlier half of the study time period TMB testing was often conducted in association with molecular testing, it is possible that patients who are younger, female, and never smokers are over represented compared to the overall NSCLC population.

## MATERIALS AND METHODS

### Patients

This was a multisite retrospective cohort study comprised of academic and community cancer centers throughout the U.S that assessed TMB and clinical outcomes among NSCLC patients at. Patients had primary malignancy of metastatic (stage IV) NSCLC, were diagnosed between 2012 and 2019 who had TMB testing and received treatment for NSCLC with at least 60 days of follow-up. The study protocol included patients with both metastatic NSCLC and small-cell lung cancer (SCLC), but only 4% of patients across all sites had SCLC. Therefore, patients with SCLC were not included in this manuscript. Results from any TMB testing platforms were included in this study.

In July 2018, academic and community cancer centers throughout the U.S. were queried by email to participate in the study. Interested sites completed a feasibility survey to report their ability to provide patient-level data. Nine sites (described in the results section) were selected based on their estimated number of patients who met study inclusion criteria and ability to collect required data elements. Following site selection, a standardized case report form and study protocol were sent to all participating sites. The study team conducted training sessions on data collection and data transfer with each participating site. Data collected from the first ten patients at each site were reviewed by the study team for quality and face validity before data extraction was completed for the remaining patients. Quality checks were also performed on the final dataset from each site. Sites were asked to provide missing data, update ambiguous responses, and correct any negative treatment intervals (e.g., initiation of NSCLC treatment prior to diagnosis of NSCLC).

### Statistical analysis

A threshold of 10 mut/Mb was used to categorize patients as having TMB-high or TMB-low according to the TMB reported by the testing vendor. The 10 mut/Mb was chosen based on the FDA’s approval of pembrolizumab for solid tumors with TMB levels greater than or equal to 10 mut/Mb and the precedent established in previous analyses of TMB in patients with lung cancer [11, 23–26]. No primary data analysis was performed on the raw sequencing files. Mutations were captured by researchers who read the primary pathology reports. Comparisons between the TMB <10 and TMB >10 cohorts for demographic and clinical characteristics were made using chi-square, Fisher’s exact test, or Student’s *t*-test, where appropriate using a pre-specified threshold of *p* < 0.05 for statistical significance.

All genomic information reported by the TMB testing platform was collected and evaluated. The reported frequency of genomic alterations accounted for the TMB test that was used for each patient. If a gene was not covered by the TMB test a patient received, the patient was not included in the analysis for that gene. Any gene reported as altered in ≥5% of patient records was included in the analysis. Association between the frequency of individual gene alterations and TMB was evaluated using the chi-square test, and a comparison of the median TMB of the altered vs. unaltered populations was done using the Wilcoxon rank-sum test. A Benjamini-Hochberg procedure was conducted to control for the false discovery rate in the comparison of median TMB by alteration status. Association of TMB with PD-L1 expression was assessed using both Student’s *t*-test and chi-square tests.

Multivariable Cox regression models were used for evaluations of OS and PFS for patients who received first-line immunotherapy-containing regimens. Predictor variables that met a conservative threshold for significance (*p* < 0.20) when assessed individually for either OS or PFS models or were prespecified as clinically important predictors were included with TMB in the multivariable models. To account for differences in CGP testing platforms, the multivariate Cox regression models for OS and PFS included only patients tested by Foundation Medicine or Caris. While the company name is included and accounted for in this analysis, we did not account for the specific TMB test as these tests have evolved over time. The subgroup of patients tested by Foundation Medicine or Caris was selected to account for variation between testing platforms. Additional Subgroup analyses were conducted for OS and PFS in patients who received TMB testing within 60 days of treatment initiation. The requirement for TMB testing 60 days prior to or 60 days post treatment initiation ensures that the TMB results were accurate during first-line therapy and limits the confounding changes that may occur to TMB results following time or treatment. An exploratory analysis of OS and PFS by TMB as a continuous variable was also conducted.

Treatment response by overall response rate was evaluated using Student’s *t*-test. Treatment response was based on physician-documentation with complete response or partial response comprising an overall response.

## CONCLUSIONS

In this multisite study across select cancer centers in the U.S., TMB levels greater than or equal to 10 mut/Mb, when tested by Foundation Medicine or Caris within 60 days of initiation of treatment, were significantly associated with OS and PFS among patients treated with first-line immunotherapy-containing regimens. Based on the results in this study and prior research, TMB along with other biomarkers, such as PD-L1, may help identify patients more likely to benefit from first-line immunotherapy. Prospective research is warranted to validate the predictive utility of this biomarker specifically in patients with low PD-L1 expression. Lower TMB was associated with actionable genomic mutations, including *ALK* and *EGFR*. Additional biomarkers would further help to identify patients likely to benefit from immunotherapy and spare the others toxicity and cost.

## SUPPLEMENTARY MATERIALS


