# Addendum: Real-world survival analysis by tumor mutational burden in non-small cell lung cancer: a multisite U.S. study

**DOI:** 10.18632/oncotarget.28318

**Published:** 2022-12-06

**Authors:** Connor Willis, Hillevi Bauer, Trang H. Au, Jyothi Menon, Sudhir Unni, Dao Tran, Zachary Rivers, Wallace Akerley, Matthew B. Schabath, Firas Badin, Ashley Sekhon, Malini Patel, Bing Xia, Beth Gustafson, John L. Villano, John-Michael Thomas, Solomon J. Lubinga, Michael A. Cantrell, Diana Brixner, David Stenehjem

**Affiliations:** ^1^Department of Pharmacotherapy, College of Pharmacy, University of Utah, Salt Lake City, UT, USA; ^2^Department of Pharmacy Practice and Pharmaceutical Sciences, College of Pharmacy, University of Minnesota, Duluth, MN, USA; ^3^Department of Internal Medicine, Huntsman Cancer Institute at the University of Utah, Salt Lake City, UT, USA; ^4^Department of Cancer Epidemiology, H. Lee Moffitt Cancer Center and Research Institute, Tampa, FL, USA; ^5^Department of Hematology and Oncology, Baptist Health Medical Group, Lexington, KY, USA; ^6^Department of Radiation Oncology, MetroHealth Medical Center, Cleveland, OH, USA; ^7^Division of Medical Oncology, Rutgers Cancer Institute of New Jersey, New Brunswick, NJ, USA; ^8^Department of Medicine, Kenneth Norris Jr. Comprehensive Cancer Center, University of Southern California, Los Angeles, CA, USA; ^9^Precision Oncology Program, Saint Luke’s Cancer Institute, Kansas City, MO, USA; ^10^Department of Internal Medicine, Markey Cancer Center, University of Kentucky, Lexington, KY, USA; ^11^US Medical Oncology, Bristol Myers Squibb, Princeton, NJ, USA; ^12^Health Economics and Outcomes Research, Bristol Myers Squibb, Princeton, NJ, USA; ^13^Global Medical Oncology, Bristol Myers Squibb, Princeton, NJ, USA

**This article has an addendum:** This study proceeded with institutional IRB approvals at each participating cancer center. Due to the retrospective nature of this project, a waiver of informed consent was approved by each of the listed local IRBs.

Original article: Oncotarget. 2022; 13:257–270. 257-270. https://doi.org/10.18632/oncotarget.28178

