# Supplementary material for: Real-world survival analysis by tumor mutational burden in non-small cell lung cancer: a multisite U.S. study
Source: Oncotarget. 2022 Jan 31;13:257–70. doi: 10.18632/oncotarget.28178 (PMC8803368; doi:10.18632/oncotarget.28178)
Supplement: Supplementary file 1 [file oncotarget-13-28178-s001.pdf]

Real-world survival analysis by tumor mutational burden in non-small cell lung cancer: a multisite U.S. study

SUPPLEMENTARY MATERIALS

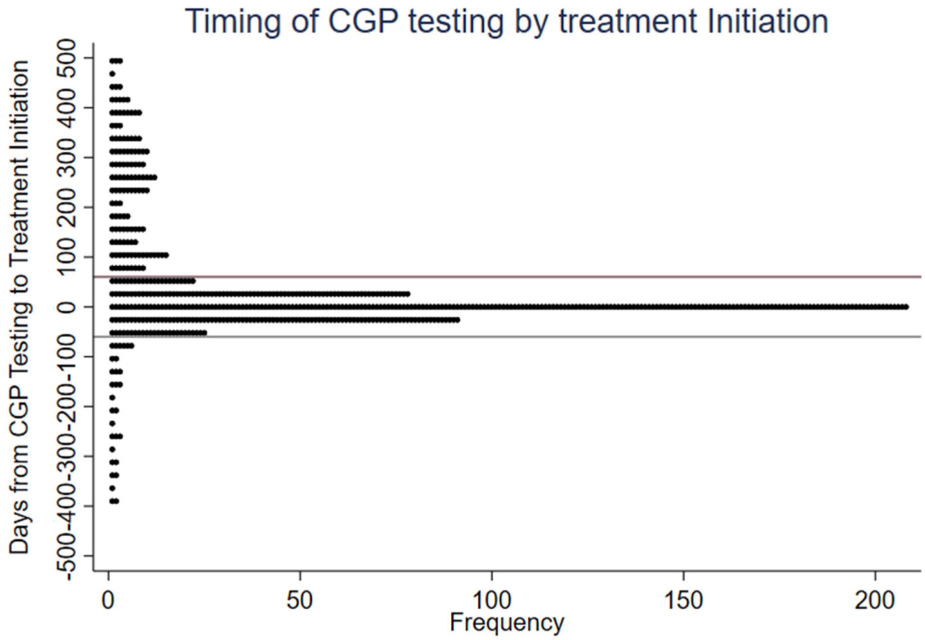

Supplementary Figure 1: Timing of comprehensive genomic profiling by treatment initiation. Reference lines are place at 60 days prior to and post initiation of first-line therapy. Patients between these reference lines were included in the subgroup analysis for OS and PFS.

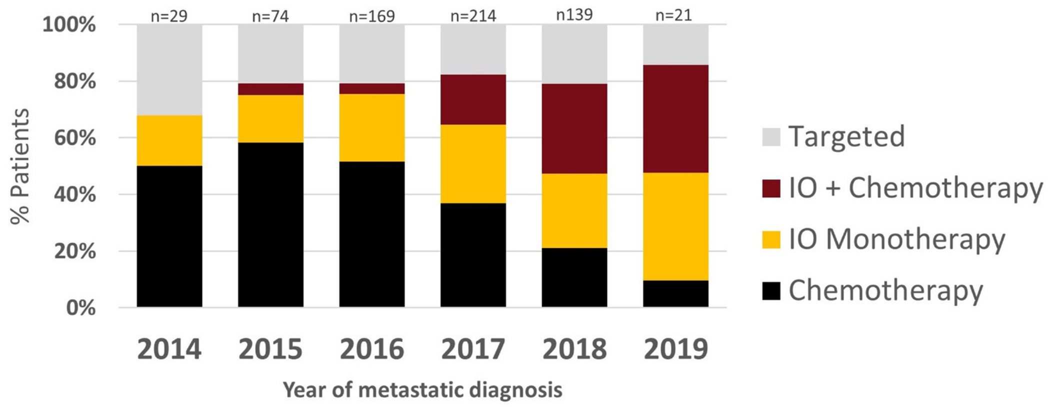

Supplementary Figure 2: First-line treatment by year of diagnosis.

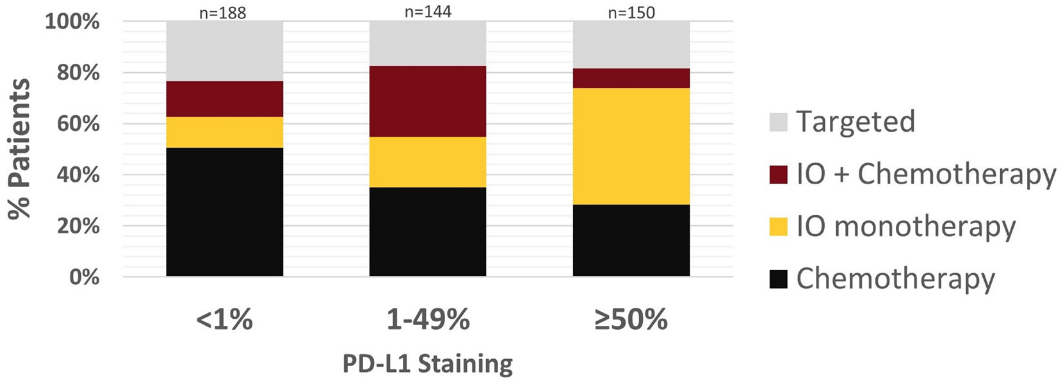

Supplementary Figure 3: First-line treatment by PD-L1 expression. Includes patients diagnosed from between Jan. 1, 2012 and Dec. 31, 2019.

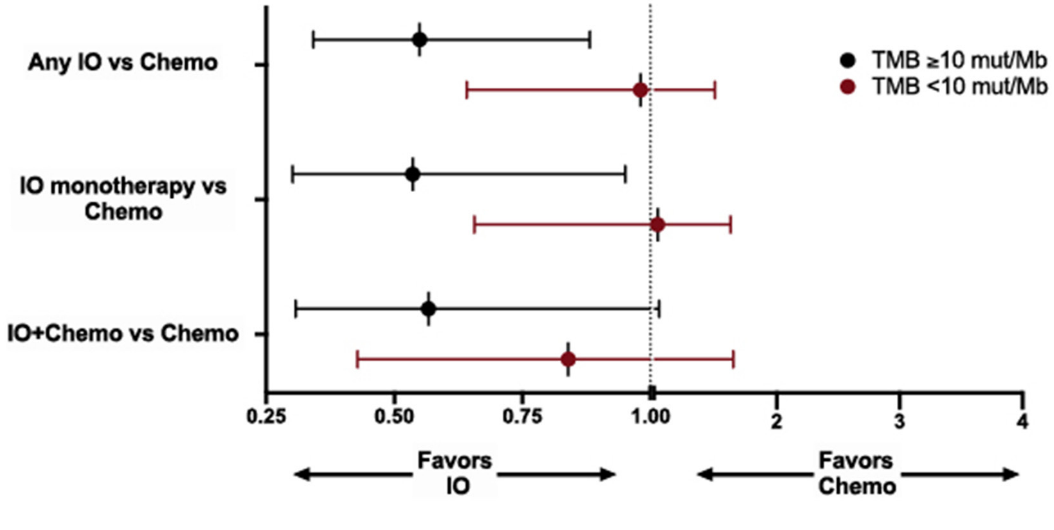

Supplementary Figure 4: Progression-free survival by treatment type and TMB.

Supplementary Table 1: TMB testing platforms

Vendor	*N* (%)	Number of genes	Depth of coverage
Foundation Medicine*	491 (64)	324–309	>500X
Caris	152 (20)	592	>750X
Oncoplex	64 (8)	339	>500X
TEMPUS*	24 (3)	648–19,396	250x–500X
NANT*	23 (3)	468–74	>500X–>100X
BioTheranostics, INC	1 (0)	36	
Cellnetix	1 (0)	13	200X
Paradigm	1 (0)	234	>5,000X
Unknown	8 (1)	NA	NA

*Multiple tests were used during the study period.
